# Seasonal atmospheric characteristics in a swine finishing barn equipped with a continuous pit recirculation system using aerobically treated manure

**DOI:** 10.5713/ab.22.0111

**Published:** 2022-06-24

**Authors:** Yongjun Choi, Duck-Min Ha, Sangrak Lee, Doo-Hwan Kim

**Affiliations:** 1 Department of Animal Science and Technology, Konkuk University, Seoul 05029, Korea; 2 Agri-Food Bio Convergence Institute, Gyeongsang National University, Jinju 52725, Korea; 3 Division of Animal Science, Agri-Food Bio Convergence Institute, Gyeongsang National University, Jinju 52725, Korea

**Keywords:** Aerated Liquid Manure, Continuous Pit Recirculation System, Indoor Atmosphere, Odorous Material, Swine Facility

## Abstract

**Objective:**

This study was conducted to determine the seasonal characteristics of odorous material emissions from a swine finishing barn equipped with a continuous pit recirculation system (CPRS) using aerobically treated manure.

**Methods:**

The CPRS consists of an aerobic manure treatment process and a pit recirculation system, where the solid fraction is separated and composted. The aerated liquid fraction (290.0%±21.0% per day of total stored pig slurry) is continuously recirculated to the top of the slurry in the pit. Four confinement pig barns in three piggery farms were used: two were equipped with CPRS, and the other two operated a slurry pit under the slatted floor across all seasons.

**Results:**

The indoor, exhaust, and outside odor intensities were significantly lower in the CPRS group than in the control group (p<0.001). In the CPRS group, the odor intensity outside was significantly lower in the fall than in the other seasons (p = 0.015). In the indoor atmosphere, the temperature and CO_2_, NH_3_, and H_2_S contents of the CPRS group were significantly lower than those of the control group (p<0.05). In the CPRS group, indoor temperature did not significantly change in the spring, summer, and fall seasons and was significantly lower in the winter (p = 0.002). NH_3_, H_2_S, methyl mercaptan, dimethyl disulfide, trimethylamine, phenol, indole, and skatole levels were significantly lower in the CPRS group than in the control group (p<0.05). There were significant seasonal differences on the odorous material in both the control and CPRS groups (p<0.05), but the pattern was not clear across seasons.

**Conclusion:**

The CPRS can reduce the indoor temperature in the summer to a level similar to that in the spring and fall seasons. The CPRS with aerated liquid manure is expected to reduce and maintain malodorous emissions within acceptable limits in swine facilities.

## INTRODUCTION

Growth and pollution emissions are closely related in most industries. In recent years, the livestock industry has been identified as a major source of pollutant emissions. All livestock excrete manure, which releases various compounds, such as, nitrogen compounds, sulfur compounds, volatile organic compounds (VOCs), and volatile inorganic compounds (VICs). Gaseous materials from livestock manure are related to air pollution and growth depression in livestock animals [1], and they contain various odorous materials. The major odorous compounds are classified into 22 types by the Malodor Prevention Act in South Korea, including ammonia (NH_3_), hydrogen sulfide (H_2_S), VOCs, phenols, sulfur-containing compounds, and volatile amines [2]. The occurrence of these odorous materials in livestock manure has resulted in an increased recognition of their negative effects in the livestock industry. In Korea, an increasing number of swine farms use continuous pit recirculation systems (CPRS) to improve indoor air quality and reduce the presence of odorous materials such as NH_3_ and H_2_S [3]. It was reported that H_2_S and NH_3_ emissions from pig finishing housing equipped with a semi-continuous pit recharge system could be reduced by 53% and 84%, respectively [3]. Furthermore, in a previous study, CPRS using aerobically treated liquid swine manure showed a reduction in various odorous materials, such as NH_3_, H_2_S, and VOCs [4]. However, in South Korea, because the CPRS system has been operating for a relatively short period, further verification is required.

In many countries, swine are raised in windowless buildings with forced ventilation. Ventilation affects the atmosphere of swine pack barns, such as temperature, humidity, and gaseous material concentration [5]. Pig houses, in particular, are ventilated differently depending on the outside environment, and their ventilation is usually controlled such that it increases as the outside temperature increases. In the CPRS system, aerobically treated liquid manure stored outside is fed to the top of the pig manure slurry to create a continuous flow. Because aerated liquid manure is stored outside, it is affected by the external environment, and the continuous flow in the pit may also affect the internal environment. To maintain the health of pigs in windowless barns, it is necessary to maintain an ideal environment. Therefore, it is necessary to investigate the changes in the environment inside pigsty equipped with a CPRS system based on the season.

Therefore, in this study, we determined the seasonal atmospheric characteristics in a swine finishing barn equipped with CPRS, using aerobically treated liquid manure.

## MATERIALS AND METHODS

### Experimental farm, continuous pit recirculation system, and experimental design

**Figure 1:**
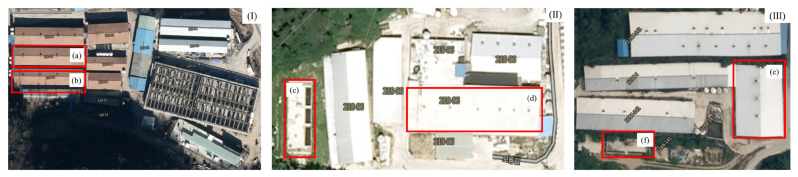
Pictures of experimental farms. (I), common farm not equipped with continuous pit recirculation system (Farm A, control); (II), Farm B; (III), Farm C; (a, b), two rooms of a pig finishing confinement building in a farm not equipped with continuous pit recirculation system; (d, e), two room of a pig finishing confinement building in the farm equipped with continuous pit recirculation system; (c, f), aerated liquid manure manufacturing facility.

We used the same pig finishing confinement building as in the previous study [4] (Table 1; Figure 1). We spent two years evaluating the effect of CPRS on the atmospheric conditions inside the pigsty. Two rooms with only a slurry pit without the CPRS system were used as a control (Table 1; Figure 1A), and two of the other rooms equipped with the CPRS system (Table 1; Figures 1B, C) were used for our experiment. The details of the rooms were presented in [4]. The CPRS system recirculates 148 kg per head of aerobically treated liquid manure daily. Recirculated aerobically treated liquid manure in a day was approximately 29.0±2.1 times per a day of the quantity of manure produced a daily by the pigs. The volume of recirculation was based on the assumption that every pig in Farms B and C excretes 5.1 kg of combined manure, urine, and wash water daily. This is the standard unit for livestock manure discharge in South Korea. The height of the slurry was maintained at a constant depth (60 cm) during the experimental periods, and the operating conditions of the CPRS system were set as detailed [4]. The recirculation process was divided into twelve steps: pit mixing, slurry out, separation, catchment, flow control, first aeration, first anoxic, second aeration, second anoxic, third aeration, settling, storage, and recirculation, according to the method of Choi et al [4]. The aerated liquid manure is called a “liquid fertilizer” and is commonly stored in a storage tank and then spread in farmlands during the periods specified by the law in South Korea. Finishing pigs were allocated to each experimental barn according to the method of Choi et al [4]. The average stocking density was 0.92±0.18 m^2^/head (Farm A, 1.00 m^2^/head; Farm B, 1.05 m^2^/head; Farm C, 0.72 m^2^/head). A fully slatted floor was installed in the barn, with 100 cm deep pits and 600 cm high shanks, and the recirculated aerated liquid manure was sampled in April, August, October, and November for 2 years at 1000 to 1200 h according to the method of Choi et al [4]. Samples were collected from the storage tank, slurry pit under the swine room, outlet of slurry pit, and anoxic tank using the method described by Choi et al [4]. Then, the temperature, humidity, and wind velocity were measured inside the barn. The air ventilation system in each room was managed independently. The air inlets were located on the ceiling, and the exhaust ventilation fans were wall-mounted (Farm A, four air inlets; Farm B, six air inlets; Farm C, three air inlets). In each room, air samples were collected from central points within the room, downstream and upstream of each continuously operated fan, and outside the room according to the method of Choi et al [4].

### Odorous materials

Air samples were collected at a height of 1.5 m using a gas sampling box (Cos-100; Kemic Co., Sungnam, Korea) equipped with a 20 L aluminum air sampling bag (TD-AP20; Whirl-Pak, Madison, WI, USA). Sampling was carried out using the same length polyvinyl chloride pipe at an inner pig finishing barn, exhaust fan, and the site boundary of the farm. Air samples were transported using a light-resistant container in the temperature range of 15°C to 25°C, and air sensory tests were conducted within 48 h after sampling. Indoor atmospheric conditions were measured inside the experimental barn with an exhaust fan using a digital anemometer assembly (Testo 410-2; Testo SE & CO., Lenzkirch, Germany) according to the method of Choi et al [4]. All odorous materials were determined according to the standard method for odor estimation in South Korea [6]. Odor intensity was scored in accordance with a 6-ladder number scale using the air dilution sensory method (the standard methods for the examination of odor [7]) in South Korea (0, undetectable; 1, barely detectable; 2, moderate; 3, strong; 4, very strong; 5, unbearable). Five expert panels determined the outcome. NH_3_ was determined by a modified colorimetric test with phenol and sodium hypochlorite [7] using a spectrophotometer (Cary 300 UV-Vis; Agilent Technologies, Santa Clara, CA, USA) at 640 nm. Sulfur compounds (H_2_S, methyl mercaptan [MM], and dimethyl disulfide [DMD]) were concentrated using a sulfur compound analyzer (Unity/Air Server XR; Markers International, Bridgend, Wales, UK) and gas chromatography (HP 6890; Agilent Technologies, USA) [8]. In this experiment, helium was used as the carrier gas and a flame photometric detector was used. Air was collected according to the impinger test method [9] for trimethylamine (TMA) analysis. The TMA was determined using a gas chromatograph equipped with a thermal desorption–cryofocusing system, according to the standard method for odor estimation in South Korea [6]. Phenol, indole, and skatole contents were determined using a gas chromatograph equipped with a solid-phase microextraction filter [10].

### Chemical analysis

Manure samples were stored at −20°C for analysis of pH, electrical conductivity (EC), biological oxygen demand (BOD), chemical oxygen demand (COD), suspended solids (SS), total nitrogen (T-N), total phosphorus (T-P), ammonium nitrogen (NH_4_-N), total organic carbon (TOC), and total carbon (TC). BOD, COD, SS, T-N, and T-P were analyzed using the standard American Public Health Association (APHA) method [11]. The pH was determined using a digital pH meter (Orion 4 Star; Thermo Scientific, Waltham, MA, USA). The EC was determined using a conductivity meter equipped with a real-time data logger (YK 2005CD; Lutron Electronic Enterprise Co., Taipei, Taiwan). The T-N content in manure was measured using the Kjeldahl method [12]. NH_4_-N was determined according to the method described by Chaney and Marbach [13]. TC and TOC were analyzed using a total organic carbon analyzer (TOC-L; Shimadzu Corporation, Kyoto, Japan).

### Statistical analysis

We analyzed the data, as a completely randomized design, using the MIXED procedure in the SAS package program (SAS Inst. Inc., Cary, NC, USA). The model was:


$${\text{Y}}_{\text{ij(t)}}=\mu +{\text{A}}_{\text{i}}+{\text{B}}_{\text{j}}+{\text{E}}_{\text{ij(t)}},$$

where μ is the average value, A_i_ is the effect of the CPRS, B_j_ is the seasonal effect, and E_ij(t)_ is the error value. The model used the CPRS and season as fixed effects. Orthogonal contrasts were used to determine the CPRS effect, seasonal effect, and interaction between the CPRS and seasonal effects using the CONTRAST option. All mean values are presented as the least-squares mean. Treatment effects were considered significant at p<0.05, and trends were considered significant at 0.05≤p<0.10.

## RESULTS

### Chemical properties of aerated manure and slurry

The seasonal variation in the chemical properties of the aerated manure recirculating in the pit of the swine barn is shown in Table 2. There was no significant seasonal difference in the EC, pH, BOD, COD, SS, T-N, T-P, NH_4_-N, TOC, and TC values of the aerated manure. The TC values were highest in spring and fall, at 3.39 and 3.61, respectively. The EC value in winter tended to be lower than the values in the spring or fall (p = 0.068). The NH_4_-N values was the lowest in winter (p = 0.096).

The chemical properties of slurry of manure pit using CPRS in the finishing pigsty (Table 3). The EC content of the CPRS group was significantly lower than that of the control group (p<0.001), and the EC contents of the control and CPRS groups did not differ significantly across seasons. The pH of the CPRS group was significantly greater than that of the control group (p = 0.034), and the pH of the control and CPRS groups did not differ significantly across seasons. The BOD, COD, SS, T-N, T-P, NH_4_-N, and TOC TC content of the CPRS group were significantly lower than those of the control group (p<0.001). The BOD, COD, SS, T-N, T-P, NH_4_-N, and TOC content showed significant differences across seasons in the control group (p<0.05), however those of CPRS group did not show significant differences across seasons.

### Odor intensity and atmospheric characteristics

The comparison of odor intensity and atmospheric characteristics in the finishing pigsty using CPRS (Table 4). The indoor, exhaust, and outside odor intensities were significantly lower in the CPRS group than in the control group (p<0.001). In the control group, the odor intensity of indoors and exhausts was significantly greater in winter than in the other seasons (p = 0.014). In the CPRS group, the odor intensity outside was significantly lower in the fall than in the other seasons (p = 0.015). In the indoor atmosphere, the temperature and CO_2_, NH_3_, and H_2_S contents of the CPRS group were significantly lower than those of the control group (p<0.05). Humidity and wind velocity did not differ between the control and the CPRS groups. In the control group, the indoor temperature was 33.1°C which was significantly higher in the summer (p = 0.001) and significantly lower in the winter (p = 0.001). The indoor temperature did not differ between the spring and fall in the control group. In the CPRS group, indoor temperature did not significantly differ among the spring, summer, and fall seasons and was significantly lower in the winter (p = 0.002). The indoor humidity of the control and CPRS groups was significantly higher in the summer and winter, respectively (p = 0.014 and p = 0.024, respectively). The indoor wind velocity in the control group was highest in the summer (p = 0.011). The CO_2_ content of the control group was significantly higher in winter (p = 0.038); there was no significant difference across the other seasons. The CO_2_ content of the CPRS group did not differ across seasons. NH_3_ and H_2_S contents did not differ across seasons in either the control or CPRS groups.[Fig f2-ab-22-0111]

### Odorous materials

A comparison of odorous material contents in the swine barn using CPRS (Table 5). NH_3_, H_2_S, MM, DMD, TMA, phenol, indole, and skatole levels were significantly lower in the CPRS group than in the control group (p<0.05). The NH_3_ and H_2_S contents did not significantly differ across seasons in either the control or CPRS groups. In the control group, the MM content was significantly higher throughout the year (p<0.001); there was no difference across seasons in the CPRS group. In the control group, DMD content was significantly higher throughout the year (p<0.001). but was not detected in the CPRS group. The TMA content of the control group was lower in the spring and winter seasons than in summer and fall seasons (p = 0.008 and p = 0.002), there was no difference across seasons in the CPRS group. Phenol content did not significantly differ across seasons in either the control or CPRS groups. In the control group, indole content was significantly higher in the winter (p = 0.051), the lowest in the spring (p = 0.037), and indole content did not differ significantly between the summer and fall seasons. In the CPRS group, indole content was significantly higher in the summer (p = 0.003), the lowest in the spring (p = 0.016), and indole content did not differ significantly between the fall and winter seasons. In the control group, skatole content was significantly higher in the season order of winter, summer, fall, and spring (p<0.05). In the CPRS group, there was no difference in the spring or across all other seasons.

## DISCUSSION

A previous study reported that CPRS can reduce odorous materials in swine barns [4]. The chemical composition of aerated liquid manure, in our study, was significantly lower than that of swine slurry [4]. However, no significant differences were observed across the seasons in the chemical composition of the aerated liquid manure, except for the TC content. Normally, the aerobic treatment process is performed under mesophilic and thermophilic conditions, and the metabolic heat of microbial fermentation maintains the temperature of the aeration process. In South Korea, the average temperature in winter is below zero, and thermal shock is the greatest inhibiting factor in microbial fermentation [14]. In this study, however, the aerobic treatment process was operated continuously, which suggests that microbial activation was maintained in the aeration tank. In addition, the aeration tank used in this study was made of concrete with a thickness of 20 cm and buried underground, which prevented temperature loss. EC and organic matter content are linearly correlated to chemical composition, such as dry matter and minerals, and they decrease as organic matter decreases during a biologically aerobic treatment process [15]. Consequently, maintaining the temperature of the aerobic treatment process is a critical factor. This can be explained by the tendency shown by the EC content (p = 0.068) and TC content (p = 0.018) being the lowest in the winter.

The chemical composition was significantly different between the control and CPRS groups. In the control group, the chemical composition of swine slurry was significantly different across seasons, except for the TC content. Generally, with increasing oxygen consumption by microorganisms during the aerobic treatment process, the BOD and COD decrease in liquid manure [16,17]. However, there was reported that BOD and COD of swine manure have high variation regardless of seasons, although there is a difference in the range of variation [18]. Therefore, it could be difficult to simply explain the difference in the BOD and COD content of the control group across the seasons in this study. Although all changes in the chemical composition of swine slurry were difficult to explain owing to the differing operation methods and ambient environment of swine farms, those using CPRS were noteworthy because the measurements did not differ across seasons. In South Korea, swine manure is mainly treated using aerobic processes within swine farms or in outside-treatment facilities. Most farms and treatment facilities lack the treatment capacity of swine manure. Because the amount of swine manure moved to the outside-treatment facilities cannot be constant, it causes serious problems with manure treatment and odorous emissions. Furthermore, it was reported that the feeding amount was a critical control point when operating aerobic treatment processes, owing to the possibility of organic loading shock [19]. Low organic loading rates are ideal for producing aerobic granules [19]. CPRS dilutes the aerated liquid manure and it shows low chemical variation across seasons. We expect that using CPRS could stabilize manure treatment in swine farms.

Owing to the change in chemical properties of the pit manure by using CPRS, the odor intensity was lower compared with the odor intensity of the control group (Table 4). It was reported that as aerobic treated liquid manure continuously flows to the upper layer of slurry in the pit, it has two effects: it dilutes the slurry with high organic matter content and blocks the odor generated from the surface of the slurry [4,20]. The odor intensity in the winter was the highest across seasons in both the control and CPRS groups, because the ventilation is reduced to maintain the temperature in the pigsty.

In the previous study, it has been shown that barn temperature and total ventilation are strongly correlated in swine confinement barns [21]. As increased total ventilation in the swine confinement barn, the air temperature inside the pig house changes similarly to the temperature outside [21]. In this study, there was no additional device to control the temperature other than the amount of ventilation in the swine confinement building. Therefore, in this study, the increase in the barn temperature shown might be explained by the influence of the external temperature due to forced ventilation. Furthermore, there was reported that air temperature a strong statistical correlated to river water temperature and flow rate [22]. The CPRS creates a continuous flow of aerated liquid swine manure to the top of the pit, it is similar to a small river. Since the temperature of the stored aerated liquid manure is lower than the outside temperature, the flow of aerated liquid manure might be one of the factors lowering the temperature inside the pig house. Pigsty equipped with CPRS showed the possibility that the thermal shock to pigs could be reduced in summer. In addition, the indoor temperature of pigsty equipped with CPRS was reasonably constant, except during the winter. In a previous study, it was reported that in thermoneutral condition (18°C to 25°C) average daily gain, feed intake, and gain to feed ratio was significantly greater than that in thermal stress conditions (25°C to 35°C) [23]. Although the indoor temperature of the CPRS group in the winter was significantly lower than that in other seasons, its temperature was within the range of the thermoneutral condition [23]. Furthermore, it was reported that a range of 0°C to 20°C did not negatively affect feed intake in swine finishing barn [24]. A previous study mentioned the possibility that CPRS could control the temperature in swine barns; this effect was demonstrated in this study [4]. Thus, CPRS is a relevant method to control the temperature in a closed swine barn regardless of the season.

In the indoor conditions, the results for CO_2_, NH_3_, and H_2_S contents were similar to those of odor intensity. Odor intensity is affected by various odorous materials in the air, and it is positively correlated between individual odorous materials and odor intensity [7]. CPRS has a positive effect on the reduction of odorous materials. However, there was minimal effect of seasonal variations on the change in odor materials. Although the odorous material content showed significant differences across seasons in the control group (Tables 3, 4), it should not be concluded that these results were due to seasonal effects. This is because odorous materials can change based on farm practices. For example, microorganisms generate the most odorous materials in animal guts and slurry pits [25], and more sulfur compounds are generated under anaerobic conditions [26]. The pit system, which is generally used in swine farms in South Korea, stores swine slurry for sufficient periods leading to anaerobic conditions depending on the storage time. This situation causes a serious odor problem owing to the low threshold of generation of odorous materials such as sulfur compounds. This problem could be rectified by storing the slurry outside the barn. However, in South Korea, this problem is not easy to solve because of the lack of livestock manure processing facilities and the small size of the land. This affects the frequency of pit cleaning; these variables cannot be controlled during the experimental period in a commercial farm. Thus, we concluded that the variation in odorous materials, in the control group, across seasons occurred due to the accumulation of uncontrollable factors in the experimental farms. However, it is important to focus on the effect of CPRS on the reduction of odorous materials and demonstrate a low variation of odorous materials across seasons. The unexpected variables of the control group are not a focus of this study. There was a similar effect of NH_3_ reduction regardless of season by using CPRS [4], that could be explained by a decrease in the T-N and NH_4_-N content in the slurry [20]. As NH_3_ and H_2_S show high solubility in water [27], there is an acceptable reduction of NH_3_ and H_2_S using CPRS. Methyl mercaptan and DMD are sulfur compounds that show low solubility in water [28]. The odorous material reduction effect of CPRS could be for two reasons. One is to inhibit anaerobic microbes, that produce sulfide compounds, by recirculating aerated liquid manure into the slurry pit [25], and the second is to block odorous material emission from slurry because of the flow of the aerated liquid manure at the top of the slurry. Indole is a signaling molecule that plays an important role in microbial communities and can affect various microbial activities such as antibiotic tolerance, spore formation, cell division, and plasmid stability [29]. Indole and skatole have low solubility in water, and studies on the degradation of indole by aerobic bacteria during composting was reported [30]. The results showed that using CPRS the reduction ratio of sulfur compounds, indoors, was greater than that of indole compounds (Table 4). It is possible that the blocking effect is greater than the supply effect of aerobic microorganisms. This study demonstrated that by using CPRS, we can reduce various odorous materials from swine slurry as well as control the indoor temperature.

## CONCLUSION

A CPRS can effectively reduce odorous materials in a swine barn, regardless of the seasonal effect. Furthermore, CPRS can decrease the indoor temperature in the summer to a similar temperature observed during the spring and fall seasons. Although the indoor temperature in winter was lower than that of a farm not equipped with CPRS, it was not in a range that would cause cold stress in pigs. The CPRS with aerated liquid manure could be expected to continuously reduce odorous materials in swine facilities and can help maintain the odor below the detection threshold.

## Figures and Tables

**Figure 2 f2-ab-22-0111:**
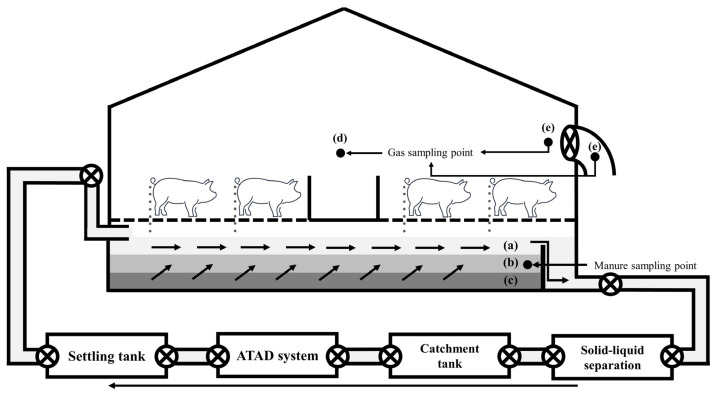
Schematic diagram of the experimental farm facilities with a continuous pit recirculation system. (a) 40 to 60 cm; (b) 20 to 40 cm; (c) under 0 to 20 cm; (d) indoor gas sampling point; (e) exhaust fan sampling point.

**Table 1:** Conditions of experimental swine houses

Farm^1)^	Total number of pigs	Housing type	Ventilation	Number of pigs in the barn	Housing size (m)	Fermenter size (m)	Storage tank (m)	Catchment tank (m)
A	4,000	Windowless	Forced	600	50×12×2.7	18×8×2	42×13×2	-
		Windowless	Forced	600	50×12×2.7	18×8×2	42×13×2	-
B	3,000	Windowless	Forced	600	35×18×4	21×8×3	7×7×4.5	30×7×3
C	2,000	Windowless	Forced	400	22×13×3	24×9×3	25×2×3	12×7×3

1) A, Common farm not equipped with a continuous pit recirculation system; B and C, farms operating continuous pit recirculation systems (Figure 1).

**Table 2:** Chemical properties of aerated liquid manure recirculating in the pit of swine barn (seasonal)

Items	Spring	Summer	Fall	Winter	SEM	p-value
EC (dS/m)	12.90	11.87	12.50	10.33	1.64	0.068
pH	7.80	7.83	7.83	7.87	0.09	0.958
BOD (g/L)	1.45	1.36	1.39	1.32	0.14	0.925
COD (g/L)	3.19	2.72	3.25	2.78	0.44	0.516
SS (g/L)	2.42	2.35	2.14	2.72	0.64	0.931
T-N (g/L)	1.09	1.00	1.05	0.90	0.09	0.428
T-P (g/L)	0.10	0.11	0.09	0.13	0.01	0.155
NH_4_-N (g/L)	0.49	0.44	0.48	0.31	0.05	0.096
TOC (g/L)	2.05	1.70	2.09	0.84	0.68	0.302
TC (g/L)	3.39^a^	2.70^ab^	3.61^a^	2.04^b^	0.48	0.018

SEM, standard error of the mean; EC, electrical conductivity; BOD, biochemical oxygen demand; COD, chemical oxygen demand; SS, suspended solids; T-N, total nitrogen; T-P, total phosphate; TOC, total organic carbon; TC, total carbon.

a,b Means within each row with different superscripts were significantly different from each other (p<0.05).

**Table 3:** Comparison of chemical properties of slurry of manure pit according to the application of a continuous pit recirculation system in the finishing pigsty (seasonal)

Items	Control				CPRS				SEM	p-value^1)^						

										CPRS	In control			In CPRS		
			
	Spring	Summer	Fall	Winter	Spring	Summer	Fall	Winter			C1	C2	C3	C1	C2	C3
EC (dS/m)	29.70	28.00	24.70	24.45	13.37	12.70	12.93	11.13	2.20	<0.001	0.594	0.134	0.937	0.797	0.867	0.492
pH	7.69	7.69	7.69	7.68	7.73	7.97	7.90	8.07	0.15	0.034	0.994	0.981	0.981	0.195	0.347	0.347
BOD (g/L)	12.73	6.47	18.73	12.93	1.58	1.30	1.76	1.84	0.56	<0.001	<0.001	<0.001	<0.001	0.675	0.788	0.907
COD (g/L)	23.19	11.43	19.95	10.33	3.50	2.98	3.70	3.02	0.81	<0.001	<0.001	0.016	<0.001	0.586	0.839	0.485
SS (g/L)	33.83	31.92	8.17	10.73	3.31	3.10	3.24	3.80	0.96	<0.001	0.185	<0.001	0.085	0.853	0.950	0.620
T-N (g/L)	5.42	5.38	2.92	4.39	1.20	1.14	1.17	1.17	0.43	<0.001	0.951	0.002	0.033	0.911	0.951	0.991
T-P (g/L)	0.97	0.81	0.37	0.36	0.15	0.13	0.15	0.17	0.06	<0.001	0.070	<0.001	0.916	0.833	0.944	0.744
NH_4_-N (g/L)	2.56	1.91	1.79	1.88	0.62	0.58	0.62	0.69	0.09	<0.001	0.000	<0.001	0.489	0.659	0.984	0.522
TOC (g/L)	14.98	9.07	9.80	7.37	2.80	2.64	2.71	1.78	0.80	<0.001	0.000	0.001	0.053	0.862	0.920	0.335
TC (g/L)	12.39	13.57	11.32	11.69	3.95	3.64	3.92	3.43	0.86	<0.001	0.354	0.401	0.767	0.762	0.978	0.633

CPRS, continuous pit recirculation system; SEM, standard error of the mean; EC, electrical conductivity; BOD, biochemical oxygen demand; COD, chemical oxygen demand; SS, suspended solids; T-N, total nitrogen; T-P, total phosphate; TOC, total organic carbon; TC, total carbon.

1) CPRS, comparison between control and CPRS groups; C1, comparison between spring and summer seasons; C2, comparison between spring and fall seasons; C3, comparison between fall and winter groups.

**Table 4:** Comparison of odor intensity and atmospheric characteristics in the finishing pigsty based on to continuous pit recirculation system (seasonal)

Items	Control				CPRS				SEM	p-value^1)^						

										CPRS	In control			In CPRS		
			
	Spring	Summer	Fall	Winter	Spring	Summer	Fall	Winter			C1	C2	C3	C1	C2	C3
Odor intensity^2)^																
Indoor	3.35	3.55	3.25	3.95	2.20	2.30	2.13	2.40	0.17	<0.001	0.426	0.688	0.014	0.623	0.742	0.203
Exhaust	3.30	3.50	3.10	4.00	2.03	2.17	1.93	2.03	0.22	<0.001	0.533	0.533	0.014	0.610	0.701	0.701
Outside	2.05	2.15	2.25	2.35	1.07	1.20	0.67	1.33	0.20	<0.001	0.735	0.502	0.735	0.582	0.115	0.015
Indoor atmosphere																
Temperature (°C)	25.9	33.1	29.1	21.0	24.2	25.3	24.3	18.9	1.2	0.000	0.001	0.092	0.001	0.442	0.909	0.002
Humidity (%)	48.4	82.1	53.3	63.6	59.9	63.9	53.4	78.2	8.3	0.711	0.014	0.685	0.399	0.685	0.510	0.024
Wind velocity (m/s)	0.00	0.65	0.15	0.00	0.03	0.13	0.27	0.17	0.15	0.620	0.011	0.499	0.499	0.580	0.209	0.580
CO_2_ (ppm)	1,273.4	1,563.7	1,626.7	2,082.4	744.7	680.3	667.0	817.7	138.0	<0.001	0.163	0.095	0.038	0.694	0.635	0.363
NH_3_ (ppm)	17.9	16.2	18.2	23.9	7.3	8.0	5.3	6.7	2.0	<0.001	0.558	0.917	0.066	0.777	0.402	0.573
H_2_S (ppm)	0.40	0.40	0.70	0.90	0.20	0.03	0.23	0.23	0.16	0.002	1.000	0.218	0.404	0.394	0.863	1.000

CPRS, continuous pit recirculation system; SEM, standard error of the means.

1) CPRS, comparison between control and CPRS group; C1, comparison between spring and summer season; C2, comparison between spring and fall season; C3, comparison between fall and winter season.

2) Odor intensity was scored according to a 6-ladder whole number scale using the air dilution sensory method of the standard methods for the examination of odor [9] (0, undetectable; 1, barely detectable; 2, moderate; 3, strong; 4, very strong; 5, unbearable). Indoor, inner sampling point of finishing pigsty (Figure 2D); exhaust, exhaust fan sampling point of finishing pigsty (Figure 2E); outside, farm site boundary sampling point.

**Table 5:** Comparison of odorous material contents in the swine barn according to the application of continuous pit recirculation system by the seasons

Items^2)^	Control				CPRS				SEM	p-value^1)^						

										CPRS	In control			In CPRS		
			
	Spring	Summer	Fall	Winter	Spring	Summer	Fall	Winter			C1	C2	C3	C1	C2	C3
NH_3_ (ppm)	14.00	16.10	16.80	24.00	5.37	5.23	8.27	8.03	2.706	0.001	0.621	0.513	0.116	0.956	0.254	0.924
H_2_S (ppm)	1.60	2.50	2.20	4.00	0.00	1.60	1.00	0.33	0.849	0.027	0.606	0.730	0.314	0.137	0.331	0.510
MM (ppb)	4.60	11.50	6.30	24.10	0.03	0.07	0.00	0.03	0.119	<0.001	<0.001	<0.001	<0.001	0.580	0.580	0.580
DMD (ppb)	1.10	1.30	1.20	0.00	nd	nd	nd	nd	-	<0.001	<0.001	<0.001	<0.001	-	-	-
TMA (ppb)	0.02	0.04	0.03	0.04	0.01	0.01	0.01	0.01	0.002	<0.001	0.008	0.184	0.002	0.426	1.000	0.871
Phenol (ppb)	6.10	7.10	6.40	6.70	4.00	3.90	2.50	2.23	2.858	0.008	0.688	0.904	0.904	0.944	0.311	0.852
Indole (ppb)	3.50	5.80	4.80	7.30	0.37	2.83	2.13	1.80	0.983	<0.001	0.051	0.230	0.037	0.003	0.016	0.580
Skatole (ppb)	0.30	3.90	3.20	7.90	nd	1.57	1.30	0.27	1.563	<0.001	0.007	0.021	0.002	0.028	0.056	0.114

CPRS, continuous pit recirculation system; SEM, standard error of the mean; MM, methyl mercaptan; DMD, dimethyl disulfide; TMA, trimethylamine; nd, not detected.

1) CPRS, comparison between control and CPRS group; C1, comparison between spring and summer season; C2, comparison between spring and fall seasons; C3, comparison between fall and winter seasons.

2) Odorous materials sampling point of finishing pigsty at indoor (Figure 2D).
